# Ten years analysis of stillbirth in a tertiary hospital in sub-Sahara Africa: a case control study

**DOI:** 10.1186/s13104-017-2787-2

**Published:** 2017-09-06

**Authors:** Paul Nkemtendong Tolefac, Rita Frinue Tamambang, Eugene Yeika, Lawrence Tanyi Mbwagbaw, Thomas Obinchemti Egbe

**Affiliations:** 1 Service of Obstetrics and Gynaecology, Douala General Hospital, Douala, Cameroon; 2 Intern Faculty of Medicine and Biomedical Sciences, University of Yaoundé 1, Yaoundé, Cameroon; 3 Saint Elizabeth General Hospital Shishong Kumbo, Kumbo, Cameroon; 4 Faculty of Health Sciences, University of Buea, Buea, Cameroon

**Keywords:** Still birth, Determinants, Case control study

## Abstract

**Objective:**

Stillbirth measures provide means to assess adequacy of maternal and perinatal care in a given population. The aim of this study was to describe the determinants of stillbirth in Douala general hospital, Cameroon.

**Results:**

Determinants of stillbirth in this hospital are: maternal age ≥35 years (OR 1.79, 95% CI 1.26–2.54, p = 0.001), pre-eclampsia/eclampsia (OR 2.97, 95% CI 0.87–8.89, p value of 0.03), diabetes in pregnancy (OR 9.97, 95% CI 1.15–86.86, p = 0.03), stillbirth in previous pregnancies (OR 3.94, CI 2.02–7.7, p < 0.0001), inter-pregnancy interval >2 years (OR 2, 06 CI 1.22–3.49; p = 0,006), referral from another hospital (OR 14.16, 95% CI 7.08–28.3, p < 0.0001), gestational age <37 (OR 19.9, 95% CI 12.3–32.2, p < 0.0001) and >42 (OR 6.27, 95% CI = 0.86–45.2, p = 0.096), congenital malformation (OR 11.09, 95% CI 3.2–38,5, p < 0.0001) and birth weight <2500 g (p < 0.0001).

**Electronic supplementary material:**

The online version of this article (doi:10.1186/s13104-017-2787-2) contains supplementary material, which is available to authorized users.

## Introduction

According to World Health Organization (WHO) stillbirth refers to the number of babies born per year with no signs of life weighing ≥1000 g and after 28 completed weeks of gestation or measuring ≥35 cm [1]. International classification of diseases version 10 (ICD-10) recommends including the number of deaths fetuses born ≥22 weeks of gestation or weighing ≥500 g as stillbirth [2]. Despite the increasing attention on maternal, neonatal and child health, stillbirth often go unrecorded and remains a major public health problem as the problem was neither considered in the millennium development goals nor in the recent sustainable development goals [3]. The burden of stillbirth is highest in developing countries as they account for 98% of the total 3.3 million stillbirths which occur annually [4]. Recently, efforts using existing data systems have been made to evaluate stillbirth rates, by modelling to obtain standard gestational age and birthweight cut-offs. Stanton et al. estimated a stillbirth rate of 25.5 per 1000 births for developing countries in the year 2000, with sub-Sahara Africa representing the highest rate (32.2 per 1000 births or a total of 889,697), followed by South Asia (31.9 per 1000 or a total of 1,286,231 births) [5]. Recent studies conducted in Nigeria estimated the stillbirth rate between 22.4 and 127/1000 [3, 6, 7]. In Cameroon, the subject is still underexploited despite increasing government attention on maternal, neonatal and child health. Two studies done earlier in the city of Yaoundé were limited to intrapartum foetal death [8, 9]. These studies estimates intrapartum foetal death rate at 1.8% [8].

Several studies carried out in Africa showed the following factors to be significantly associated with stillbirth when compared with live births: multiparty, antepartum haemorrhage, premature rupture of membranes, hypertensive conditions in pregnancy, caesarean section, cephalopelvic disproportion, prolonged/obstructed labour, and congenital abnormalities, young maternal age, lack of antenatal care, Primary or no maternal education labour complications, home delivery birth weight less than 2500 g, cord accidents, and foetal distress [6, 10–12].

There have not been any study on stillbirth in Douala general hospital (DGH) and Cameroon at large. Available studies in Cameroon have concentrated on intrapartum foetal death and neonatal mortality [8, 9]. The main aim of the study was to assess sociodemographic, medical and obstetric determinants of stillbirth in the maternity of DGH.

## Main text

### Methods

#### Study design and setting

A 1:2 unmatched case control study was carried out in the maternity of Douala general hospital. A case was any mother who had a stillbirth in the maternity of DGH from 1st January 2006 to 31st December 2015. A control was a live birth in the same maternity during the same period. For every case of stillbirth, we selected two control delivered either 3 days before or after in the same maternity.

DGH is a 1st level tertiary hospital located in the economic capital of Cameroon, Douala. It is one of the fastest growing hospitals in the central African sub region. The maternity is well equipped with modern equipment and personnel to provide comprehensive Emergency Obstetric and Neonatal Care (EmONC) services.

#### Data collection and analysis

Using a 95% confidence interval, minimum power to detect a difference of 80%, and a hypothetical ratio of control to cases of 2:1 and assuming a minimum odd ratio of 2 for differences to be detected based on previous studies with similar designs [10], we estimated the minimum sample size at 534 women comprising 178 mothers of stillbirth babies (cases) and 356 mothers of live birth (controls) using Fleiss formula online calculator [13]. All cases within the 10 years period were included and controls chosen at a ratio of 1 case:2 controls. The controls were chosen during the same time period we had a case of stillbirth that is for every case of stillbirth we chose a control delivered 3 days before or after. Structured questionnaire was administered to collect sociodemographic data, obstetric history and medical history. The data was coded and double entered into a predesigned template in *Epi Data* then exported to SPSS version 20.0 for analysis. We compared percentages between two groups using Chi square test. Odd ratios, 95% confidence intervals and p values were estimated to identify associations between independent variables (predictor) and stillbirth (outcome). Significance level was set at a p value less than 0.05.

#### Definition of terms

Still birth was defined in this study as a death of a foetus weighing ≥500 g occurring antepartum after 22 weeks of gestation. A case was defined in our study as any stillbirths recorded in the maternity of DGH and controls were live births recorded in the maternity within the same period.

### Results

9738 women delivered in the maternity of DGH between 1st January 2006 and 31st December 2015. There were a total of 251 stillbirths and we selected 492 controls (live births) given a ratio of approximately 1:1, 9. The mean age of the study population was 31 years with a range of 17–44 years.

#### Sociodemographic determinants

Maternal age ≥35 years (OR 1.79, 95% CI 1.26–2.54, p = 0.001), being married (OR 1.49, 95% CI 1.09–2.06, p = 0.016) and referral from another hospital (OR 14.16, 95% CI 7.08–28.3, p < 0.0001) were sociodemographic factors found to be associated with stillbirth (Table 1).

**Table 1:** Sociodemographic determinants of stillbirth

Variables	Category	Cases	Controls	OR (CI 95%)	p value
		N (%)	N (%)		
Maternal age	<35 years	175 (69.7)	396 (80.5)	1	
	≥35 years	76 (30.3)	96 (19.5)	1.79 (1.26–2.54)	0.001
Marital status	Single	96 (38.2)	144 (29.3)	1	
	Married	155 (61.8)	348 (70.7)	1.49 (1.09–2.06)	0.016
Level of education	Primary	123 (49)	193 (39.2)		
	Secondary	26 (10.4)	59 (12)	1	
	University	102 (40.6)	240 (48.8)	0.96 (0.57–1.61)	0.9
Referral status	From home	194 (77.3)	482 (98.0)	1	
	From another hospital	57 (22.7)	10 (2.0)	14.16 (7.08–28.3)	<0.0001
Profession	Medical personnel	7 (2.8)	18 (3.7)	1	
	Secretary	82 (32.7)	127 (25.8)	1.66 (0.66–4.15)	0.28
	Accountant	31 (12.4)	56 (11.4)	1.42 (0.54–3.78)	0.48
	Teacher/lecturer	19 (7.6)	34 (6.9)	1.44 (0.51–4.06)	0.49
	Housewife	61 (24.3)	93 (18.9)	1.69 (0.66–4.28)	0.27
	Technician	22 (8.8)	49 (10.0)	1.15 (0.42–3.16)	0.78
	Student	29 (11.6)	115 (23.4)	0.65 (0.25–1.70)	0.38

#### Medical determinants

Medical conditions associated with stillbirth as shown in Table 2 included: diabetes in pregnancy (OR 9.97, 95% CI 1.15–86.86, p = 0.03) pre-eclampsia/eclampsia (OR 2.97, 95% CI 0.87–8.89, p value of 0.03).

**Table 2:** Medical determinants of stillbirth

Variable	Response	Cases	Controls	OR (CI 95%)	p value
		N (%)	N (%)		
Alcohol consumption	No	233 (92.8)	466 (94.7)	1	
	Yes	18 (7.2)	26 (5.3)	1.38 (0.74–2.57)	0.3
Smoking	No	251 (100.0)	491 (99.8)	1	
	Yes	0 (0)	1 (0.2)		0.7
Chronic hypertension	No	246 (98.0)	490 (99.6)	1	
	Yes	5 (2.0)	2 (0.4)	4.97 (0.95–25.8)	0.08
Pre-eclampsia/eclampsia	No	244 (97.2)	487 (99.0)	1	
	Yes	7 (2.8)	5 (1.0)	2.79 (0.87–8.89)	0.12
Diabetes in pregnancy	No	246 (98.0)	491 (99.8)	1	
	Yes	5 (2)	1 (0.2)	9.97 (1.15–86.86)	0.03
Obesity	No	245 (97.6)	487 (99.0)	1	
	Yes	6 (2.4)	5 (1.0)	2.38 (0.72–7.89)	0.25
HIV	No	236 (94.0)	474 (96.3)	1	
	Yes	15 (6.0)	18 (3.7)	1.67 (0.82–3.37)	0.2

#### Obstetrics determinants

As shown on Table 3 (Additional file 1), obstetric determinants associated with stillbirth in our maternity included: history of stillbirth in the previous pregnancies (OR 3.94, CI 2.02–7.7, p < 0.0001), inter-pregnancy interval greater than 2 years (OR 2, 06 CI 1.22–3.49; p = 0.006), gestational age <37 weeks (OR 19.9, 95% CI 12.3–32.2, p < 0.0001) and >42 weeks (OR 6.27, 95% CI 0.86–45.2, p = 0.096), caesarean delivery (OR 1.96, 95% CI 1.34–2.93, p = 0.0008), congenital malformation (OR 11.09, 95% CI 3.2 – 38,5, p < 0.0001), birth weight <2500 g (p < 0.0001).

**Table 3:** Obstetric determinants of stillbirth

Variable	Response	Cases	Controls	OR (CI = 95%)	p value
		N (%)	N (%)		
History of stillbirth	No	225 (89.6)	478 (97.2)	1	
	Yes	26 (10.4)	14 (2.8)	3.94 (2.02–7.7)	<0.0001
IUGR	No	251 (100.0)	491 (99.8)		
	Yes	0 (0.0)	1 (0.2)		
Spontaneous abortion	No	209 (83.3)	415 (84.3)	1	
	Yes	42 (16.7)	77 (15.7)	1.08 (0.71–1.63)	0.78
Interpregnancy interval	≤2	30 (12.0)	100 (20.3)	1	
	>2	57 (22.7)	92 (18.7)	2.06 (1.22–3.49)	0.006
	No interval	164 (65.3)	300 (61.0)		
Parity	Prim-parous	78 (31.1)	157 (31.9)	1	
	Pauci-parous	112 (44.6)	225 (45.7)	1 (0.7–1.42)	0.93
	Multi-parous	53 (21.1)	100 (20.3)	1.07 (0.69–1.63)	0.85
	Grand multi-parous	8 (3.2)	10 (2.0)	1.61 (0.61–4.24)	0.47
Number of ANCs	1–2	13 (5.2)	38 (7.7)	1	
	3–4	26 (10.4)	48 (9.8)	1.58 (0.78–3.49)	0.32
	≥5	52 (20.7)	140 (28.5)	0.92 (0.45–1.86)	0.8
	Not available	160 (63.7)	266 (54.1)	0.5 (0.29–1.1)	0.12
Gestational age	22–28	84 (33.5)	3 (0.6)	175.6 (54.1–570.8)	<0.0001
	29–36	95 (37.8)	30 (6.1)	19.9 (12.3–32.2)	<0.0001
	37–42	70 (27.9)	439 (89.2)	1	0.062
	≥42	2 (0.8)	2 (0.4)	6.27 (0.86–45.2)	0.096
	Not available	0 (0.0)	18 (3.7)		
Mode of delivery	Vaginal	192 (76.5)	426 (86.6)		
	Caesarean	59 (23.5)	66 (13.4)	1.98 (1.34–2.93)	0.0008
Birth weight	50–1500	116 (46.2)	8 (1.6)	96.2 (44.8–206.5)	<0.0001
	1500–2500	50 (19.9)	25 (5.1)	13.3 (7.7–22.9)	<0.0001
	2500–4000	63 (25.1)	418 (85)	1	0.09
	≥4000	10 (4.0)	32 (6.5)	2.07 (0.97–4.42)	0.06
	Not available	12 (4.8)	9 (1.8)		
Congenital malformations	No	235 (93.6)	489 (99.4)	1	
	Yes	16 (6.4)	3 (0.6)	11.09 (3.2–38.5)	<0.0001

Factors associated with stillbirth in a multivariate analysis (Table 4, Additional file 1) included: referral from another hospital (OR 14.86 95% CI 3.35–66.01, p = 0.0004, inter-pregnancy interval greater than 2 years (OR 3.59, 95% CI 1.29–9.98, p = 0.014), birth weight less than 2500 g: birth weight 1500–2500 (OR 6.11, 95% CI 1.81–20.59, p = 0.004), birth weight 500–1500 (OR 627.79, 95% CI 72.47–5438.14 p < 0.0001) and congenital malformations (OR 13.46, 95% CI 1.10–165.29, p = 0.042).

**Table 4:** Multivariate analysis of factors associated with stillbirth

Factor	OR	95% CI lower limit	95% CI upper limit	p value
Maternal age	1.09	0.38	3.24	0.80
Marital status	1.59	0.50	5.13	0.43
Notion of referral	14.86	3.35	66.01	0.0004
Pre-eclampsia	0.75	0.08	7.01	0.80
Past history still birth	1.42	0.22	9.35	0.72
Past history of diabetes	NA			
Interpregnancy interval >2 years	3.59	1.29	9.98	*0.014*
Preterm labour/threatened abortion	4.03	0.86	18.84	0.077
Delivery by caesarean section	2.92	1.07	7.97	0.036
Birth weight 2500–4000	0.71	1.22	5.68	0.091
Birth weight 1500–2500	6.11	1.81	20.59	0.004
Birth weight 500–1500	627.79	72.47	5438.14	<0.0001
Gestational age 37–41 weeks 6 days				0.42
Gestational age: 28–36 weeks 6 days	1.41	0.33	6.03	0.64
Gestational age: 22–27 weeks days	10.76	0.51	225.55	0.13
Congenital malformations	13.46	1.10	165.29	0.042

### Discussion

In this unmatched case control study we assessed sociodemographic, medical and obstetric determinants of stillbirths in DGH over 10 years. The results of the study revealed the following determinants: maternal age ≥35 years, referral from another hospital, diabetes in pregnancy, preeclampsia/eclampsia, history of stillbirth, inter-pregnancy interval >2 years, gestational age <37 and >42, low birth weight <2500 g and congenital malformations.

The association of maternal age greater than 35 years and stillbirth was similar to that obtained in other developing countries where increasing age was associated to stillbirth [14–16]. This could be explained by the fact that the incidence of chromosomal and genetic abnormalities in women increases with advanced maternal age. The odds of developing stillbirth were 4 times higher if the woman have had a previous stillbirth. This was equally similar to studies done in other developing countries where history of previous stillbirth was a determinant of stillbirth [10, 15]. This explains the need for close follow up in subsequent pregnancies if the woman has a history of stillbirth as the odds of repeating are higher after one stillbirth. The association between pre-eclampsia/eclampsia and stillbirth has been well described in literature as this usually leads to utero—placenta insufficiency, intrauterine growth restriction and consequently intrauterine foetal death. This association has been confirmed in numerous studies [14, 15, 17] and similarly in our study as the odds of developing stillbirth was about 3 times higher in women with a history of pre-eclampsia/eclampsia (OR 2.97, 95% CI 0.87–8.89, p value of 0.03). This association disappeared when we did a multivariate analysis (p = 0.80). The odds of developing still birth were twice as likely if the inter-pregnancy interval was greater than 2 years (OR 2.06; 95% CI 1.22–3.49; p = 0.006). Earlier studies have shown that increasing the inter-pregnancy interval increases the risk of preeclampsia/eclampsia [18] and hence stillbirth. The odds of delivering a stillbirth were 14 times higher in parturient referred compared to those that came from home to the maternity of DGH (OR 14.16; 95% CI 7.08–28.3; p < 0.0001). Previous studies had earlier confirmed this association [8, 15]. This could be explained by delayed referral, long distances, poor conditions of transportation, poor communication, lack of specialties and lack of comprehensive package to provide comprehensive Emergency Obstetric and Neonatal Care (EmONC). There was a significant association between birth weight and stillbirth, the odd of delivering a death foetus increases as the birth weight decreases, this was significant for all birth weights less than 2500 g. This has earlier been demonstrated by studies in Ghana and Kenya respectively [10, 12]. Gestational age <37 weeks (p > 0.0001) and >42 weeks (OR 6.27, 95% CI 0.86–45.2) increases the odds of delivering a stillbirth. This was demonstrated in an earlier study in Kenya [12]. This could be explained by the fact the more remote the gestational age is from term, the greater the risk of prematurity and its complications including low birth which we earlier demonstrated in this study to be associated with stillbirth and further by the fact that as the pregnancy advances further from a term of 37–42 weeks, placental insufficiency increases and hence the stillbirth rate. The odds of having a baby with congenital malformation were 11 times in parturients with stillbirths compared with those with live births (OR 11.09; 95% CI 3.2–38.5; p < 0.0001). This has been demonstrated in earlier studies [14]. Also Charlotte et al. [19] in 2015 in a study on congenital malformations in the same maternity, demonstrated that the babies of parturients with congenital malformations were more likely to have stillbirth.

#### Recommendations to policy makers and future researchers

Earlier studies in 2015 by Halle et al. [20] in the same maternity showed that 64% of maternal deaths were referred cases from other health institutions and that in about 80% of the referral, the referring institution did not respect the referral system in Cameroon [20]. This has been confirmed by our study as patients who came from another institution had higher odds of having stillbirths compared to patients who came from home. We therefore recommend to district and private hospitals referring patients do DGH and other tertiary structures to refer on time and use the appropriate means available. We also recommend to the ministry of health to document the existing referral system in Cameroon and educate health facilities on its proper use and implementation. To future researchers, we recommend that multicentre case control studies and or a systematic review and meta-analysis.

### Conclusion

Determinants of stillbirth in our series of patients included: advanced maternal age ≥35 years, inter-pregnancy interval >2 years, referral from other sanitary structures, low birth weight <2500 g, gestational age less than 37 weeks and greater than 42 weeks, congenital malformations, caesarean section. Other determinants in the past history were history of diabetes, preeclampsia/eclampsia and previous stillbirth. Adequate prevention and management of identified risk factors constitute major steps in the reduction of the number of stillbirth by upgrading and equipping all maternities with a minimum package for emergency obstetrics and neonatal care.

## Limitations

Some important limitations to the study include:

- Information not present on all determinants because of it retrospective nature.
- Most stillbirths still occur at home.
- The study was conducted only in one centre in sub-Sahara in an urban area.

## Additional file

**Additional file 1.** Case report form.

## Abbreviations

IUGR: intrauterine growth restriction
DGH: Douala General Hospital
EmONC: emergency obstetric and neonatal care
ICD: International classification of diseases
ANC: antenatal care
WHO: World health organisation
