# Supplementary material for: Ten years analysis of stillbirth in a tertiary hospital in sub-Sahara Africa: a case control study
Source: BMC Res Notes. 2017 Sep 6;10:447. doi: 10.1186/s13104-017-2787-2 (PMC5585898; doi:10.1186/s13104-017-2787-2)
Supplement: Supplementary file 1 — Additional file 1. Case report form. [file 13104_2017_2787_MOESM1_ESM.docx]

**CASE REPORT FORM**

**TITLE: Ten years analysis of stillbirth in a tertiary hospital in sub-Sahara Africa: a case control study**

**Principal investigator**: Dr Paul N. TOLEFAC

**CONFIDENTIAL INFORMATION**

Woman’s Name: _______________________________________

Medical file’s code: __ __ __/ __

**Medical file’s Code: __ __ __ / __**

| **S/N** | **Question** | **Choices** | **Respond** |
| --- | --- | --- | --- |
| **I** | **Sociodemographic characteristics** | | |
| SIQ01 | Date of birth |  |  |
| SIQ02 | Born | [1] Alive  [2] Death |  |
| SIQ03 | Age of women |  |  |
| SIQ04 | Marital status | [1] married  [2] single  [3] divorce |  |
| SIQ05 | Level of education | [1] no formal education  [2] primary  [3] secondary  [4] university |  |
| SIQ06 | Profession | [1] Medical personnel  [2]Secretary  [3] Accountant  [4] Teacher / lecturer  [5] Housewife  [6] Technician  [7] Student  [8] Other____________ |  |
| SIQ07 | Referral Status | [1] From home  [2] From another hospital |  |
| II | **Medical history** | | |
| SIIQ08 | Alcohol consumption | [1] Yes  [2] No |  |
| SIIQ09 | Smoking | [1] Yes  [2] No |  |
| SIIQ10 | Chronic hypertension | [1] Yes  [2] No |  |
| SIIQ11 | Pre – eclampsia / eclampsia | [1] Yes  [2] No |  |
| SIIQ12 | Diabetes in pregnancy | [1] Yes  [2] No |  |
| SIIQ13 | Obesity | [1] Yes  [2] No |  |
| SIIIQ14 | HIV | [1] Yes  [2] No |  |
| III | **Obstetric history** | | |
| SIIIQ15 | Gravidity |  |  |
| SIIIQ16 | Total parity |  |  |
| SIIIQ17 | Gestational age at delivery |  |  |
| SIIIQ18 | ANC | [1] Yes  [2] No |  |
| SIIIQ19 | Number of ANCs |  |  |
| SIIIQ20 | Interpregnancy interval |  |  |
| SIIIQ21 | Birthweight |  |  |
| SIIIQ22 | History of stillbirth | [1] Yes  [2] No |  |
| SIIIQ23 | IUGR | [1] Yes  [2] No |  |
| SIIIQ24 | Spontaneous abortion | [1] Yes  [2] No |  |
| SIIIQ25 | Congenital malformation present at birth | [1] Yes  [2] No |  |
| SIIIQ26 | Mode of delivery | [1] Vaginal  [2] caesarean |  |
